# Development and Validation of a Novel Serum Prognostic Marker for Patients with Metastatic Colorectal Cancer on Regorafenib Treatment

**DOI:** 10.3390/cancers13205080

**Published:** 2021-10-11

**Authors:** Yu-Li Su, Kai-Lung Tsai, Tai-Jan Chiu, Yueh-Ming Lin, Ko-Chao Lee, Chien-Chang Lu, Hong-Hwa Chen, Chia-Che Wu, Hung-Chih Hsu

**Affiliations:** 1 Division of Hematology Oncology, Department of Internal Medicine, Kaohsiung Chang Gung Memorial Hospital, College of Medicine, Chang Gung University, Kaohsiung 833, Taiwan; chiutaijan@gmail.com (T.-J.C.); wu.chiache@msa.hinet.net (C.-C.W.); 2 Clinical Trial Center, Kaohsiung Chang Gung Memorial Hospital, Kaohsiung 833, Taiwan; 3 Department of Colorectal Surgery, Kaohsiung Chang Gung Memorial Hospital, College of Medicine, Chang Gung University, Kaohsiung 833, Taiwan; kltsai@cgmh.org.tw (K.-L.T.); alcohol@cgmh.org.tw (Y.-M.L.); kclee@cgmh.org.tw (K.-C.L.); doctor.lu@msa.hinet.net (C.-C.L.); ma2561@cgmh.org.tw (H.-H.C.); 4 Division of Hematology Oncology, Chang Gung Memorial Hospital at Linkou, College of Medicine, Chang Gung University, Taoyuan 333, Taiwan

**Keywords:** metastatic colorectal cancer, regorafenib, neutrophil to lymphocyte ratio, CEA, overall survival, liver metastasis, predictive model

## Abstract

**Simple Summary:**

Regorafenib has proven its efficacy for later-line treatment of mCRC. However, treatment often brings substantial toxicities that lead clinicians to assess the risk-to-benefit ratio in heavily pretreated patients. Thus, it is crucial to develop a prognostic factor and model for guiding patient selection. In this study, we represent a new serum biomarker to serve as an independent prognostic factor for patients receiving regorafenib. All 4 factors of the prognostic model were employed with an excellent discriminatory ability. This result should be validated in further confirmatory studies.

**Abstract:**

(1) Background: To investigate the prognostic value of cancer-inflammation prognostic index (CIPI) in patients with metastatic colorectal cancer (mCRC) on regorafenib treatment; (2) Methods: Patients with mCRC who were given regorafenib as later-line treatment at Kaohsiung and Linkou Chang-Gung Memorial Hospital between November 2014 and January 2021 were consecutively enrolled. All relevant clinicopathologic, laboratory data and survival status were recorded. Independent prognostic factors were determined by the multivariate Cox regression method; (3) Results: In total, 106 patients in the training cohort and 250 in the validation cohort were enrolled. The median OS for patients with CIPI ≥ 300 and < 300 in the training cohort was 3.8 and 9.0 months, respectively (hazard ratio (HR) 2.78, 95% confidence interval (CI) 1.82–4.23; *p* < 0.0001). Time to regorafenib, liver metastasis and CIPI were independent factors by multivariate Cox regression analyses. A new scoring model demonstrated a good discriminatory ability to risk stratification of a patient’s survival; (4) Conclusions: We identified CIPI as a novel serum marker highly associated with overall survival in patients with mCRC receiving regorafenib treatment. Further confirmatory studies are warranted.

## 1. Introduction

Metastatic colorectal cancer (mCRC) is a lethal disease and was the third leading cause of cancer deaths in 2018 [1,2]. With major advances in chemotherapy, targeted therapy, and aggressive surgical resection over the past two decades, the median overall survival (OS) of patients with mCRC has been significantly prolonged from 12 to 36 months [3,4,5]. Some patients have the chance to achieve a durable survival by integrating radical surgery, chemotherapy, and targeting therapy including anti-epidermal growth factor receptor (anti-EGFR) or anti-vascular epidermal growth factor (VEGF) antibody [6]. However, most patients with mCRC eventually experience progressive disease and unendurable symptoms. Later-line therapy, such as regorafenib and trifluridine/tipiracil (TAS-102), demonstrated a modest anti-tumor efficacy and overall survival benefits compared to a placebo for chemorefractory mCRC [7,8,9,10].

Regorafenib is a potent, oral-form tyrosine kinase inhibitor that effectively blocks the angiogenic (VEGFR1/3, PDGF-b), stromal (FGFR1), and some driver oncogenic kinases (KIT, RET and BRAF) [11]. The pivotal CORRECT study demonstrated that patients on regorafenib treatment had a significant survival benefit compared with those on placebo treatment (6.4 months vs. 5.0 months; hazard ratio 0.77; 95% CI 0.64–0.94) [8]. The efficacy of regorafenib for mCRC in third-line treatment was confirmed by several real-world studies [12,13]. The CORRELATE study designed as a prospective, observational study that aimed to evaluate the real-world safety and efficacy of regorafenib disclosed a similar toxic profile and disease control rate to that in the CORRECT study [12,13]. Nevertheless, only a small fraction of patients benefit from regorafenib treatment (objective response rate 1%; disease control rate 41% in CORRECT study) and that limits physicians’ willingness to use it unless a reliable predictive or prognostic biomarker is discovered.

Previous studies have identified and developed some potential predictive factors to estimate the OS on regorafenib. Novakova-Jiresova et al. reported a large retrospective series of 555 patients with mCRC on regorafenib and found 3 independent good prognostic factors by multivariate analysis: high body-mass index (BMI), longer interval from metastatic disease to regorafenib and Eastern Cooperative Oncology Group Performance Status (ECOG PS) of 0 [14]. On top of that, Del Prete et al. found that serum inflammation markers, such as high neutrophil, platelet counts and high neutrophil to lymphocyte ration (NLR), negatively influenced OS in patients receiving regorafenib [15]. A recent study constructed a prognostic model for patients who underwent regorafenib and identified that low carcinoembryonic antigen (CEA), slow rate of tumor progression, and fewer organ metastatic sites were highly correlated with good overall survival [16]. Collectively, this imperative research emphasized that a good predictive model should consider both patient (ECOG PS), tumor (CEA, numbers of metastatic site) and immune (NLR) related factors simultaneously.

Here, we introduced a novel prognostic index, defined as the cancer-inflammation prognostic index (CIPI) by calculating the values of CEA multiply NLR, that was developed and validated externally. By incorporation of this novel marker with previous established predictors, we aimed to develop a new prognostic model that makes it easy for physicians to choose patients for regorafenib wisely.

## 2. Materials and Methods

### 2.1. Patients, Data Process and Treatment

This retrospective cohort study analyzed patient data from two independent medical centers in Taiwan: Kaohsiung Chang Gung Memorial Hospital (the training cohort) and Linkou Chang Gung Memorial Hospital (the validation cohort). Patients with histologically proven mCRC and refractory to standard chemotherapy (fluorouracil, oxaliplatin, irinotecan) plus anti-VEGF or anti-EGFR therapy were enrolled for analysis. All clinicopathologic and laboratory data were retrieved from electrical medical recording (EMR) systems. Database variables included age, sex, ECOG PS, primary tumor site, RAS status, visceral organ metastasis, interval from metastasis to regorafenib administration, and serological factors (white blood cell count, hemoglobin, platelet count, neutrophil count, lymphocyte count and CEA). We collected laboratory data within 1 week prior to patients undergoing regorafenib treatment. The starting dosage of regorafenib treatment depended on a physician’s discretion considering the patient’s performance status, comorbidities, and potential adverse effects. The study was approved by the Institutional Review Board of Chang Gung Medical Foundation (201801598B0C502).

### 2.2. Response Evaluation and Endpoints

All patients on regorafenib therapy had been regularly scheduled for a clinic visit and evaluated for treatment response by CT scans of the chest or abdomen or serologic tumor markers. As application of regorafenib reimbursement required the latest CT images to prove therapeutic efficacy, patients had to receive a CT scan every 8 weeks, and that ensured the window of radiographic evaluation was similar to that in the CORRELATE study. The assessment of treatment response was using the criteria of Response Evaluation Criteria in Solid Tumors (version 1.1). The primary endpoint of this study was OS, which was defined as the interval between the date of initiating regorafenib treatment and the date of death.

### 2.3. Cancer-Inflammation Prognostic Index (CIPI)

We defined CIPI by calculating the following equation: CIPI = CEA × NLR, where NLR represents the ratio of neutrophil and lymphocyte count. To obtain the optimal cutoff value of CIPI, we used X-tile 3.6.1 software (Yale University, New Haven, CT, USA) for analysis of data from the training cohort [17]. The receiver operating characteristic (ROC) curve analysis was employed to evaluate the distinguish ability of CIPI and the prognostic model.

### 2.4. Statistics

All statistical analyses were performed using SPSS version 25.0 (SPSS Inc., Chicago, IL, USA) and R software 4.1.1. Data visualization and depiction of survival curves was plotted using GraphPad Prism version 8.21 (GraphPad Software, La Jolla, CA, USA). Descriptive analysis of all clinicopathological variables of the training and validation cohorts were examined by using chi-squared (χ^2^) and *t* tests for categorical and continuous variables, respectively. Estimates of overall survival were determined by the Kaplan–Meier method and examined for group differences statistically with the log-rank test. Univariate and multivariate analyses of independent factors were performed using the Cox proportional hazards regression analysis. A *p*-value < 0.05 was considered statistically significant.

## 3. Results

### 3.1. Patient Characteristics

In total, 356 eligible patients were incorporated into the final analysis, including 106 patients in the training cohort and 250 patients in the validation cohort. The median age of all patients was 61 years (interquartile range (IQR), 53–68 years), and the median follow-up time was 20.7 months. Among all, 210 (59%) patients were men, and 74.7% of patients had a left-sided tumor. The differences of demographic and clinical features between the training and validation cohorts are shown in Table 1. Except for ECOG performance status (*p* = 0.01) and the initial dose of regorafenib (*p* < 0.001), all other characteristics were compared without significant difference.

### 3.2. Determine the Cutoff Level of CIPI

**Figure 1:**
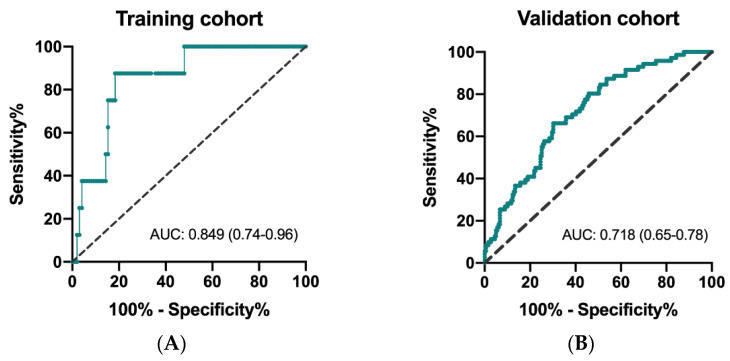
Receiver operating characteristic (ROC) curve for CIPI and mortality in the training cohort (**A**) and in the validation cohort (**B**).

The ROC curves to determine predictivity of CIPI and overall survival are depicted for the training and validation cohorts. As shown in Figure 1A,B, the area under curve (AUC) was 0.849 (95% CI: 0.74–0.96; *p* = 0.001) in the training cohort and 0.718 (95% CI: 0.65–0.78; *p* < 0.0001) in the validation cohort, respectively. The results of X-tile analysis disclosed that the optimal cutoff level of CIPI in the training cohort was 302.0 (maximum high/low chi-square = 24.3955, Miller–Seigmund *p* < 0.0001). We defined the recommended cut-off value of CIPI as 300 for further validation analysis.

### 3.3. Univariate and Multivariate Analysis of OS and PFS in the Training Cohort

**Figure 2:**
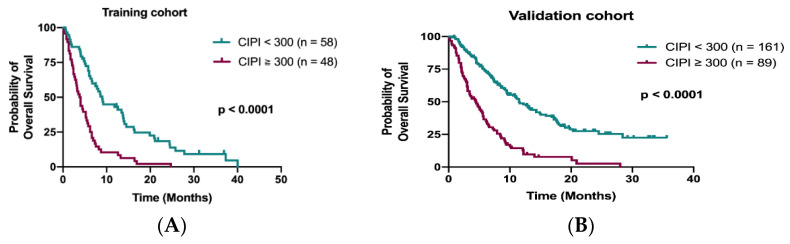
Kaplan–Meier curve for OS stratified by CIPI at 300 in the training cohort (**A**) and in the validation cohort (**B**).

Data from the training cohort were used to identify prognostic factors and to build up a predictive model. A total of 98 out of 106 patients (92.5%) died during the follow-up period. The median OS in the training cohort was 6.0 months (95% confidence interval (CI), 4.7–7.3 months). The Kaplan–Meier analysis demonstrated that patients with CIPI ≥ 300 had a significantly worse OS than those with CIPI < 300 (3.8 months vs. 9.0 months; *p* < 0.0001; Figure 2A). The other significant prognostic factors in the training cohort from the univariate analysis were ECOG PS, time to regorafenib treatment, primary tumor resection, hemoglobin, visceral metastasis (liver, bone and peritoneum) and initial dose of regorafenib (Table 2). Multivariable Cox regression analyses of these factors and OS from the training cohort disclosed that time to regorafenib treatment < 24 months (HR 2.27; 95% CI, 1.44–3.60; *p* < 0.0001), liver metastasis (HR 1.61; 95% CI, 1.00–2.64; *p* = 0.05) and CIPI ≥ 300 (HR 2.14; 95% CI, 1.23–3.74; *p* = 0.007) determined OS significantly (Table 2).

**Figure 3:**
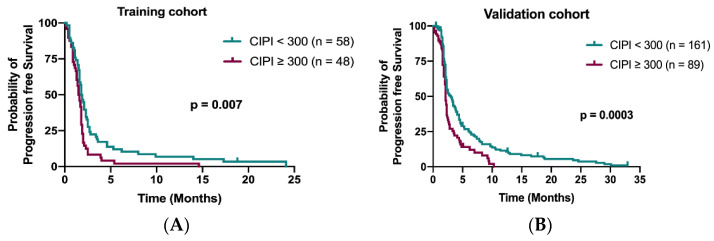
Kaplan–Meier curve for PFS stratified by CIPI at 300 in the training cohort (**A**) and in the validation cohort (**B**).

The median PFS in the training cohort was 1.7 months (95% confidence interval (CI), 1.58–1.91 months). Patients with CIPI < 300 demonstrated a better PFS than those with CIPI ≥ 300 in the training cohort (1.8 months vs. 1.5 months; *p* = 0.007; Figure 3A) and in the validation cohort (3.0 months vs. 2.1 months; *p* = 0.0003; Figure 3B). Multivariable Cox regression analyses demonstrated that time to regorafenib treatment < 24 months (HR 1.76; 95% CI, 1.15–2.68; *p* = 0.009) and peritoneum metastasis (HR 2.18; 95% CI, 1.34–3.56; *p* = 0.002) were significant independent factors for PFS (Table 3).

### 3.4. Build Up and Validation of the Prognostic Model

**Figure 4:**
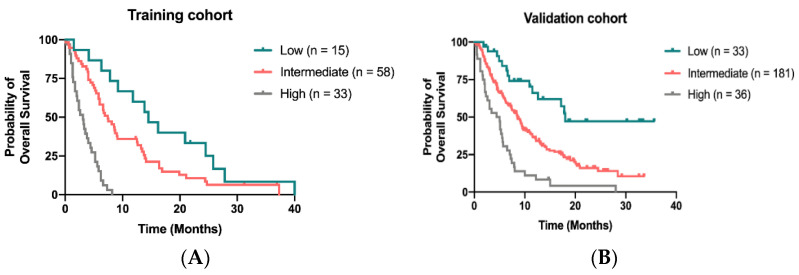
Kaplan–Meier curve for OS stratified by the scoring model in the training cohort (**A**) and in the validation (**B**).

We developed a novel prognostic model to predict OS for patients receiving regorafenib by calculating 4 independent factors: time to regorafenib treatment < 24 months, liver metastasis, peritoneal metastasis and CIPI ≥ 300. By attributing one point for each risk factor and summed up for a total score, patients with scores of 0, 1–2 and 3–4 were classified as low-, intermediate-, and high-risk groups, respectively. The median OS was 14.5 months (95% CI, 8.9–20.1 months) 7.4 months (95% CI, 5.5–9.3 months) and 3.1 months (95% CI, 2.1–4.1 months) for patients in low-, intermediate- and high-risk groups, respectively (Figure 4A). In the validation cohort, the median OS for patients assigned to low, intermediate and high-risk groups was 18.0 months, 8.6 months and 4.4 months, respectively (Figure 4B).

## 4. Discussion

Regorafenib is approved globally as a recommended drug in the third-line treatment for mCRC [18,19]. Although the survival benefit for patients on regorafenib reached significance statistically, only a small fraction of patients responded to regorafenib. Given the substantial toxicity from regorafenib and frailty of chemorefractory patients, it is crucial for physicians to balance treatment efficacy and consequent adverse effects. Identification of a predictive biomarker to guide decision making is becoming a fundamental aspect of clinical practice. Our results confirmed that CIPI has a good discriminatory power in predicting mortality for patients who underwent regorafenib. In combination with the other poor prognostic factors, we developed a new scoring model that can separate curves of OS nicely according to different risk groups. We believe this model can help physicians to make clinical decisions wisely.

Several exploratory studies that aimed to identify the reliable molecular marker of regorafenib failed to reach consensus because they lacked validation and reproducibility. In the CORRECT study, Tabernero et al. demonstrated that KRAS and PIK3CA mutation status determined by BEAMing analysis of circulating tumor DNA (ctDNA) were not correlated with regorafenib efficacy [20]. In addition to tumor genomic alteration, the study also analyzed many serum proteins aimed at finding therapeutic correlation. High serum concentrations of TIE1 were associated with longer overall survival than low serum TIE1 in univariate analyses, but did not show significance in the multivariate analyses [20]. The exploratory analysis of 16 plasma proteins concentration and OS in the CONCUR study also failed to identify any potential candidate, suggesting that plasma protein level was not suitable for predicting regorafenib efficacy [21].

CEA has been routinely employed as a surrogate marker to detect early recurrence for patients with localized CRC who underwent surgical resection [22]. For advanced mCRC, the level of CEA has shown an inverse correlation with poor survival despite patients receiving therapy aggressively [23,24,25]. Soluble CEA can elicit proangiogenic endothelial cell adhesion and migration, and it enhances tumor neovascularization independent of the VEGF signaling pathway [26]. In the subgroup analysis of the RAISE study, a low level of baseline CEA (cutoff 10 ng/mL) was a decisive factor to differentiate patients who gained benefits from ramucirumab and chemotherapy combination [25]. Prager et al. also reported the predictive role of baseline CEA only for bevacizumab-based therapy, not for cetuximab and chemotherapy combination [27]. However, data of the CEA level were not recorded and analyzed in the prospective large trials (CORRECT, CONCUR and REBECCA), leading to no convincing evidence to understand the predictive role of baseline CEA for regorafenib therapy [8,9,28]. A recent study enrolled a total of 613 patients to explore the predictive clinicopathologic factors for mCRC on regorafenib treatment [16]. Hsu et al. disclosed that serum CEA level was inversely correlated with the OS of patients receiving regorafenib. Our findings demonstrated consistent results, suggesting that the level of circulating CEA has a critical role in determining the OS for patients on regorafenib therapy.

Cancer is well-recognized as a chronic inflammation disease. Cancer-associated inflammation is a complex phenomenon that involves numerous circulating blood cells, chemokines, stromal cells and metabolic factors [29]. It is known that leukocytes, particularly neutrophils, play a crucial role in tumor microenvironment promoting cancer cell proliferation, invasion, metastasis and resistance to chemotherapy [30,31,32,33]. Elevated absolute neutrophil count (ANC) was proven as a strong predictor for treatment outcome in patients receiving curative surgery or systemic chemotherapy [34,35]. The predictive and prognostic role of NLR has been extensively discussed in CRC and other solid cancers [36,37,38]. Several studies investigated the prognostic role of NLR in patients treated with regorafenib. Del Prete et al. found that several serum markers such as high LDH levels, neutrophil, platelet counts and high NLR were negatively correlated to OS [15]. Our data demonstrated similar results: high NLR (cutoff at 4) determining OS powerfully in the univariate Cox regression analysis (HR 2.01; 95% CI 1.30–3.10). Although NLR is undoubtedly a robust surrogate marker of overall survival, the hurdle of clinical utilization is lacking a definitive cutoff level of NLR. Further studies and more data are needed to come up with the ideal level of NLR.

One of the impressive findings in this study is that the combination of CEA with NLR as a new predictor (CIPI) contributed highly to the risk stratification of patients’ survival. Our predictive model, by counting only 4 factors (CIPI, time to regorafenib, liver and peritoneum metastasis), has a good discrimination effect to risk stratification of survival. Several prognostic models have been developed and implemented in clinical practice. The “Colon Life” nomogram raised by Pietrantonio et al. consisted of simple 4 clinical factors: ECOG PS, primary tumor resection, LDH and peritoneal metastasis [39]. The nomogram discriminative ability in both the developing and validating set were excellent (Harrell C index 0.778). However, despite the fact that ECOG PS < 2 and primary tumor resection are associated with a favorable survival in the univariate analysis of our patients, the prognostic significances were not shown in the multivariate Cox regression model. We believe a comprehensive model covering all aspects including patient, tumor and therapy has the best predictive value for patients with mCRC who are treated with regorafenib.

There are several limitations in this study. First, the nature of retrospective design has a hidden selective bias. Because the study lacks a control group, it is better to claim CIPI is a prognostic rather than a predictive marker. Second, toxicity from regorafenib was not reported in the retrospective study because many constitutional symptoms and non-hematologic abnormal data were not recorded in medical charts. Third, the initial dosage of regorafenib was not uniform. Heavily pretreated patients with mCRC may not be suitable for a standard dosage of regorafenib. Dose escalation strategy, such as the ReDDoS study protocol, is commonly used in real-world practice [40]. Lastly, our real-world data are based on a small sample size included in only two medical centers. Further research with a larger sample size may provide better homogeneity and validity.

## 5. Conclusions

In summary, the results of the present study identified CIPI as a robust and easy to use clinical factor for patients with mCRC who are receiving regorafenib. The new scoring model by integrating CIPI, time to regorafenib, liver and peritoneal metastasis demonstrated an excellent discriminant validity of OS. Further big sample-size studies are needed to confirm our findings.

## Figures and Tables

**Table 1:** Clinical characteristics of the training and validation cohorts.

Characteristic	All (*n*, %)	Training Cohort (*n*, %)	Validation Cohort (*n*, %)	*p*-Value
*n*	356	106	250	
Age (mean, SD), years	60.38 ± 11.2	62.02 ± 10.3	59.69 ± 11.5	
Median (range)	61 (17–97)	64 (39–83)	60 (17–97)	
Sex				
Female	146 (41.0)	43 (40.6)	103 (41.2)	0.99
Male	210 (59.0)	63 (59.4)	147 (58.8)	
ECOG PS				
0–1	332 (93.3)	93 (87.7)	239 (95.6)	0.01
≥2	24 (6.7)	13 (12.3)	11 (4.4)	
Primary site				
Left	266 (74.7)	75 (70.8)	191 (76.4)	0.17
Right	83 (23.3)	30 (28.3)	53 (21.2)	
Multifocal	7 (2.0)	1 (0.9)	6 (2.4)	
Primary tumor resection				
Yes	299 (84.0)	91 (85.8)	208 (83.2)	0.64
No	57 (16.0)	15 (14.2)	42 (16.8)	
RAS status				
Wild	175 (49.2)	47 (44.3)	128 (51.2)	0.2
Mutant	177 (49.7)	59 (55.7)	118 (47.2)	
NA	4 (1.1)	0	4 (1.6)	
MMR status				
Proficient MMR	154 (43.3)	55 (51.9)	99 (39.6)	0.09
Deficient MMR	6 (1.7)	2 (1.9)	4 (1.6)	
NA	196 (55.1)	49 (46.2)	147 (58.8)	
Organ metastasis				
Liver	196 (55.1)	64 (60.4)	132 (52.8)	0.2
Lung	226 (63.5)	70 (66.0)	156 (62.4)	0.55
Bone	34 (9.6)	8 (7.5)	26 (10.4)	0.55
Peritoneum	64 (18.0)	24 (22.6)	40 (16.0)	0.17
Previous treatment				
Anti-VEGF	340 (95.5)	103 (97.2)	237 (94.8)	0.41
Anti-EGFR	168 (47.2)	49 (46.2)	119 (47.6)	0.82
Time to regorafenib (month)				
≥24	180 (50.6)	50 (47.2)	130 (52.0)	0.42
<24	176 (49.4)	56 (52.8)	120 (48.0)	
NLR				
<4	241 (67.7)	71 (67.0)	170 (68.0)	0.9
≥4	115 (32.3)	35 (33.0)	80 (32.0)	
Hemoglobin (g/dL)				
≥10	278 (78.1)	83 (78.3)	195 (78.0)	0.99
<10	78 (21.9)	23 (21.7)	55 (22.0)	
Initial dose of Regorafenib				
mean ± SD	133.8 ± 30.7	103.8 ± 23.3	146.6 ± 23.7	<0.001
160 mg	186 (52.2)	5 (4.7)	181 (72.4)	
120 mg	108 (30.3)	53 (50.0)	55 (22.0)	
80 mg or less	62 (17.5)	48 (45.3)	14 (5.6)	
CEA, median, (ng/mL)	53.1	62	47.9	0.15
CEA (ng/mL)				
<50	173 (48.6)	46 (43.4)	127 (50.8)	0.21
≥50	183 (51.4)	60 (56.6)	123 (49.2)	

Abbreviations: CEA, carcinoembryonic antigen; ECOG PS, Eastern Cooperative Oncology Group performance status; EGFR, epidermal growth factor receptor; MMR, mismatch repair; NLR, neutrophil to lymphocyte ratio; RAS, rat sarcoma virus; VEGF, vascular endothelial growth factor; SD, standard deviation.

**Table 2:** Univariate and multivariate analyses for OS in the training cohort.

	Univariate		Multivariate	
Variables	HR (95% CI)	*p*	HR (95% CI)	*p*
Sex (male vs. female)	0.92 (0.61–1.38)	0.67		
Age, years (≥60 vs. <60)	0.71 (0.47–1.09)	0.11		
ECOG PS (≥2 vs. 0–1)	2.42 (1.30–4.52)	0.004	1.40 (0.69–2.87)	0.36
Primary site (right vs. left)	1.02 (0.65–1.59)	0.95		
Primary tumor resection (no vs. yes)	2.45 (1.39–4.31)	0.001	0.85 (0.42–1.71)	0.65
RAS status (MT vs. WT)	1.19 (0.79–1.78)	0.41		
Time to regorafenib, mo (<24 vs. ≥24)	1.91 (1.27–2.89)	0.002	2.27 (1.44–3.60)	<0.0001
Liver metastasis (yes vs. no)	2.02 (1.33–3.07)	0.001	1.61 (1.00–2.64)	0.05
Lung metastasis (yes vs. no)	0.93 (0.61–1.43)	0.75		
Bone metastasis (yes vs. no)	2.77 (1.32–5.81)	0.005	1.66 (0.73–3.78)	0.23
Peritoneum metastasis (yes vs. no)	1.87 (1.13–3.09)	0.013	1.68 (0.96–2.93)	0.07
NLR (≥4 vs. <4)	2.01 (1.30–3.10)	0.001		
Hemoglobin, g/dL (<10 vs. ≥10)	2.25 (1.37–3.69)	0.001	1.42 (0.79–2.56)	0.24
CEA, ng/mL (≥50 vs. <50)	1.93 (1.27–2.94)	0.002		
Initial dose of regorafenib ^¶^ (low vs. high)	1.75 (1.16–2.64)	0.007	1.48 (0.94–2.33)	0.09
CIPI (≥300 vs. <300)	2.78 (1.82–4.23)	<0.0001	2.14 (1.23–3.74)	0.007

Abbreviation: CEA, carcinoembryonic antigen; CI, confidence interval; ECOG PS, Eastern Cooperative Oncology Group performance status; EGFR, epidermal growth factor receptor; HR, hazard ratio; NLR, neutrophil to lymphocyte ratio; MT, mutant type; RAS, rat sarcoma virus; VEGF, vascular endothelial growth factor; WT, wild type. ^¶^ High dose: 120 or 160 mg; low dose: 40 or 80 mg.

**Table 3:** Univariate and multivariate analyses for PFS in the training cohort.

	Univariate		Multivariate	
Variables	HR (95% CI)	*p*	HR (95% CI)	*p*
Sex (male vs. female)	1.07 (0.61–1.38)	0.73		
Age, y (≥60 vs. <60)	0.72 (0.48–1.08)	0.10		
ECOG PS (≥2 vs. 0–1)	1.82 (1.01–3.29)	0.04	1.14 (0.61–2.15)	0.68
Primary site (right vs. left)	0.98 (0.64–1.52)	0.94		
Primary tumor resection (no vs. yes)	1.56 (0.90–2.72)	0.11		
RAS status (MT vs. WT)	1.12 (0.76–1.65)	0.57		
Time to regorafenib, month (<24 vs. ≥24)	1.74 (1.16–2.60)	0.006	1.76 (1.15–2.68)	0.009
Liver metastasis (yes vs. no)	1.66 (1.10–2.48)	0.01	1.52 (0.94–2.45)	0.09
Lung metastasis (yes vs. no)	0.75 (0.50–1.13)	0.17		
Bone metastasis (yes vs. no)	1.31 (0.63–2.70)	0.47		
Peritoneum metastasis (yes vs. no)	2.10 (1.31–3.37)	0.001	2.18 (1.34–3.56)	0.002
NLR (≥4 vs. <4)	1.65 (1.08–2.50)	0.02		
Hemoglobin, g/dL (<10 vs. ≥10)	1.68 (1.05–2.70)	0.03	1.36 (0.83–2.23)	0.23
CEA, ng/mL (≥50 vs. <50)	1.31 (0.88–1.94)	0.17		
Dose of regorafenib ^¶^ (low vs. high)	1.04 (0.70–1.54)	0.84		
CIPI (≥300 vs. <300)	1.69 (1.14–2.51)	0.007	1.24 (1.77–1.98)	0.38

Abbreviation: CEA, carcinoembryonic antigen; CI, confidence interval; ECOG PS, Eastern Cooperative Oncology Group performance status; EGFR, epidermal growth factor receptor; HR, hazard ratio; NLR, neutrophil to lymphocyte ratio; MT, mutant type; RAS, rat sarcoma virus; VEGF, vascular endothelial growth factor; WT, wild type. ^¶^ High dose: 120 or 160 mg; low dose: 40 or 80 mg.

## Data Availability

The data present in this study are available from the corresponding author upon request.
